# Mapping chromosomal regions associated with anther indehiscence with exerted stigmas in CRI-48 and Jasmine 85 cross of rice (*Oryza sativa* L)

**DOI:** 10.1016/j.heliyon.2021.e06483

**Published:** 2021-03-13

**Authors:** Samuel Oppong Abebrese, Nana Kofi Abaka Amoah, Paul Kofi Ayirebi Dartey, Isaac Kofi Bimpong, Richard Akromah, Vernon Edward Gracen, Samuel Kwame Offei, Eric Yirenkyi Danquah

**Affiliations:** a CSIR-Savanna Agricultural Research Institute, P.O. Box TL 52, Tamale, Ghana; b Africa Rice Centre, Headquarters, M'bé Research Station. 01 B.P 2551, Bouaké o1, Cote d’Ivoire; c CSIR-Crops Research Institute, P. O. Box 3785, Fumesua, Ghana; d Department of Crop and Soil Sciences, KNUST, Kumasi, Ghana; e West Africa Centre for Crop Improvement, University of Ghana, Legon, Ghana

**Keywords:** Outcrossing, Genetic mapping, Hybrid rice seed production, SNP markers, Pleiotropic gene action

## Abstract

Anther indehiscence in certain wide crosses combines male sterility with stigma exertion, a phenomenon that is desirable for hybrid rice seed production. This study sought to identify chromosomal region(s) that combine anther indehiscence with exerted stigmas. A mapping population consisting of 189 BC_1_F_1_ plants was derived from a cross between CRI-48 and Jasmine 85 and backcrossing the resulting F_1_ to Jasmine 85. Contrary to the three complementary genes mode of inheritance reported earlier, a single locus (*AI6-1*) was mapped on chromosome 6 at 27.4 cM for anther indehiscence with exerted stigmas through a mixed model-based composite interval mapping (MCIM). This locus was flanked by two single nucleotide polymorphism (SNP) markers, K_ID6002884 and K_ID6003341 within a range of 23.1–28.9 cM. The allele at the locus was contributed by the CRI-48 parent which has *Oryza glaberrima* ancestry. This locus is suggested to control anther indehiscence and stigma exertion through pleiotropic gene action or cluster of genes.

## 1 Introduction

Rice (*Oryza sativa* L.) is a major staple food crop in the developing world (Guimaraes, 2009; Seck et al., 2012). It is cultivated on 11% (156 million ha) of the world's total arable land second only to wheat in terms of harvested area (FAO, 2017). The demand for rice globally, is predicted to increase as a result of increased growth in population (IRRI, 2010; Seck et al., 2013; Muthayya et al., 2014). Khush (2005) estimates that global production will have to increase by 40% by the year 2030 to meet the growing demand for rice. Genetic improvement of rice has led to significant yield increases; however, average yields of inbred varieties have reached a plateau making further increments difficult (Khush, 2005; IRRI, 2010; Khan et al., 2015). Hybrid technology which exploits the phenomenon of heterosis presents a viable means of significantly increasing rice yield than the semi-dwarf inbred varieties currently being utilised (IRRI 1997; Guimaraes, 2009; Fischer et al., 2014; Khan et al., 2015).

Rice, being a strictly self-pollinating crop requires the use of a male sterility system to develop commercial hybrid varieties (Virmani, 1994; Virmani et al., 2003). Cytoplasmic male sterility (CMS) and environment-conditioned genetic male sterility (EGMS) are the two male sterility systems currently available for hybrid rice seed production. The extent and scope of outcrossing determine the ability of these male sterility systems to increase the efficiency of hybrid seed production. Earlier studies have indicated that efficiency of cross pollination in rice is influenced by floral traits including flowering behaviour, pollen longevity, stigma exertion and spikelet opening angle (Virmani, 1994; Takano-Kai et al., 2011). Among these, stigma exertion is the most important trait since it is directly involved in pollination (Virmani, 1994; Takano-Kai et al., 2011; Lou et al., 2014; Bakti and Tanaka, 2019; Xu et al., 2019).

Anther indehiscence, resulting from certain wide crosses, has been suggested as a form of functional male sterility (Sano, 1986; Oka, 1991; Maekawa et al., 1997; Dartey, 2007; Abebrese et al., 2018) with different modes of inheritance (Cheng and Huang 1980; Sano, 1986; Tamaru, 1991; Maekawa et al., 1997; Dartey, 2007). It has been found to combine male sterility with stigma exertion in specific crosses, a phenomenon believed to adapt the indehiscent plants to outcrossing (Dartey, 2007; Abebrese et al., 2018). This unique combination of anther indehiscence and stigma exertion could present a perfect male sterility system for hybrid rice seed production. The exerted stigmas would trap more pollens from the male parent thereby reducing the pollination barrier often encountered with some cytoplasmic male sterile lines and would increase hybrid seed set (Virmani, 1994; Takano-Kai et al., 2011).

Recent advances in molecular marker technology through quantitative trait loci (QTL) analysis, allow the identification of chromosomal region(s) underlying important traits in plants (McCouch and Doerge, 1995; Young, 1994; Toure et al., 2000; Collard et al., 2005; Jones et al., 1997, 2009; Nadeem et al., 2018). Breeders can get an insight into the number of loci controlling a trait, their relative importance and approximate positions in the genome (Jones et al., 1997, 2009; Breseghello and Coelho, 2013; Nadeem et al., 2018). Several marker systems are currently available for QTL mapping in plants (Semagn et al., 2006; Collard et al., 2008; Jones et al., 2009; Nadeem et al., 2018). Among these, single nucleotide polymorphism (SNP) markers have emerged as the marker of choice due to their low assay costs, high genomic abundance, locus-specificity, co-dominant inheritance, potential for high throughput analysis and relatively low rates of genotyping error (Semagn et al., 2006, 2014; McCouch et al., 2010, 2013). The continuous progress in high-throughput genomic technologies has led to numerous SNP genotyping platforms that combine a variety of chemistries and allele discrimination techniques (Semagn et al., 2014; Nadeem et al., 2018). Among these is the kompetitive allele specific PCR (KASP) (LGC group); a homogenous fluorescence-based genotyping variant of polymerase chain reaction which works based on allele-specific oligo extension and fluorescence resonance energy transfer for signal generation. This has emerged as a more flexible and cost-effective technique with minimal rate of genotyping error (Collard et al., 2008; Semagn et al., 2014; Smith and Moughan, 2015; Steele et al., 2018; Yang et al., 2019).

Over 20 genes have been reported to be involved in regulating anther dehiscence in plants (Keijzer, 1987; Goldberg et al., 1993; Matsui et al., 1999; Ma, 2005; Kobayashi et al., 2011; Wilson et al., 2011; Zhou et al., 2011; Peng et al., 2013; Ling et al., 2015; Cardarelli and Costantino, 2018; Estornell et al., 2018; Moon and Jung, 2020). For rice, Zhu et al. (2004) mapped anther indehiscence gene (*aid1*) on chromosome 6 using a two-element iAc/Ds transposon-tagging system. Using a similar approach, Thangasamy et al. (2011) also found that rice SUMO E3 ligase (*siz1*) gene on chromosome 5 controls spikelet fertility through regulation of anther dehiscence. Anther indehiscence in these two studies (Zhu et al., 2004; Thangasamy et al., 2011) was not associated with stigma exertion, but the genes had pleiotropic effect on other traits. Several studies have also mapped QTLs for stigma exertion on different rice chromosomes (Uga et al., 2003; Miyata et al., 2007; Yan et al., 2009; Li et al., 2014; Lou et al., 2014). Studies on the possible environmental effects on anther indehiscence with exerted stigmas suggested that light, temperature and relative humidity could not modulate the sterility/fertility status of anther indehiscence plants (Zhu et al., 2004; Abebrese et al., 2018; Estornell et al., 2018). Our earlier study (Abebrese et al., 2018) found three complementary genes mode of inheritance for anther indehiscence with exerted stigmas in the CRI-48/Jasmine 85 cross. Information on the chromosomal location of genes controlling anther indehiscence with exerted stigmas is currently lacking. Although it was previously not possible to employ nuclear controlled male sterility in hybrid rice seed production due to the inability to propagate a pure male sterile line, genetic engineering technique now allows constructing useable nuclear male sterile lines for hybrid rice seed production (Chang et al., 2016). Knowledge of the genes controlling anther indehiscence with exerted stigmas at the molecular level could help in manipulating the trait with advanced breeding techniques to develop a useable male sterility system with enhanced outcrossing for hybrid rice seed production. Therefore, as the first step, this study was carried out to identify chromosomal region(s) controlling anther indehiscence with exerted stigmas in a BC_1_F_1_ population of rice.

## 2 Materials and methods

### 2.1 Plant material

**Figure 1:**
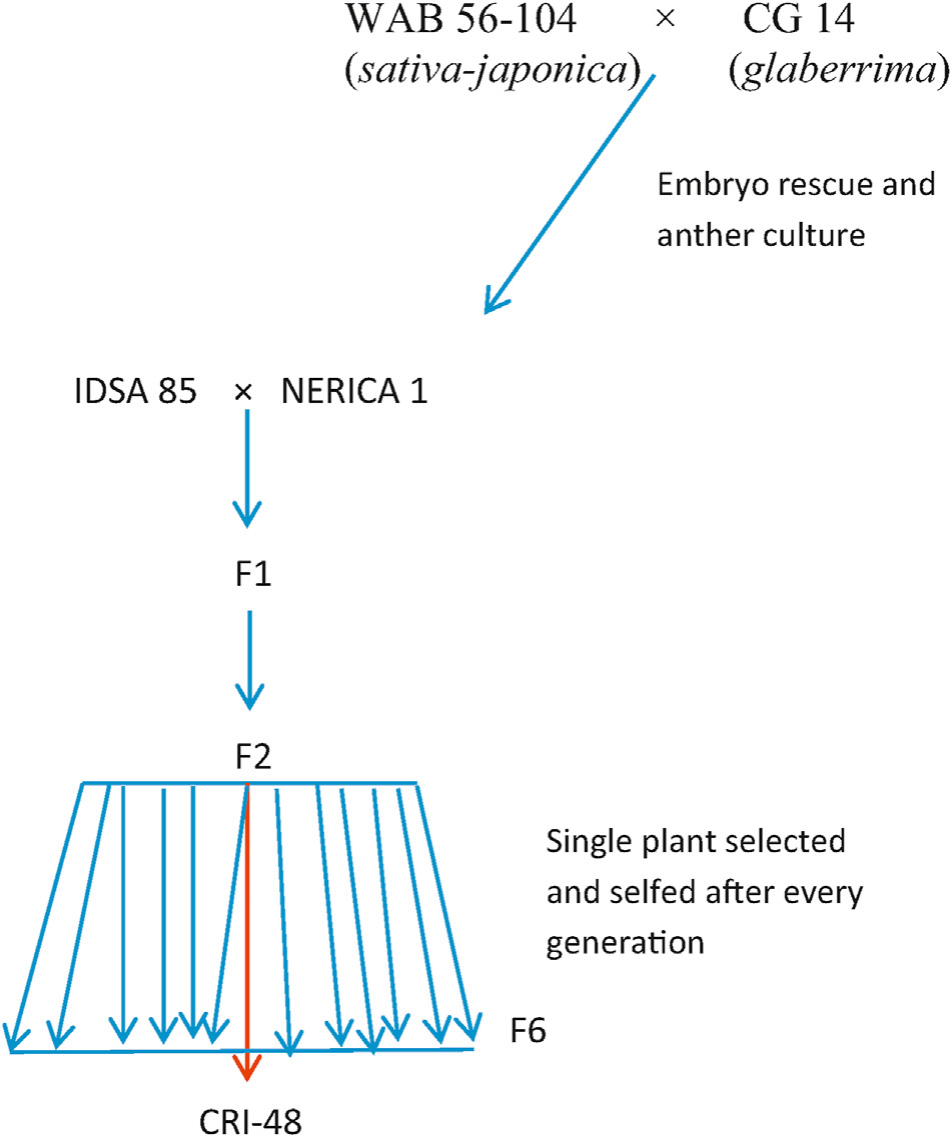
Pedigree of CRI-48 (CR-48 is a recombinant inbred line from IDSA 85 and NERICA1 cross. NERICA 1 is an interspecific line developed from WAB 56–104 (*sativa-japonica*) and CG 14 (*glaberrima*)).

The parental materials used were two elite rice genotypes, CRI-48 (female) and Jasmine 85 (male). CRI-48 is an interspecific stabilized breeding line developed at the Council for Scientific and Industrial Research - Crops Research Institute (CSIR-CRI), Fumesua, Ghana, from the cross IDSA 85 × NERICA 1 (Figure 1). It has dehiscent anthers and non-exerted stigmas. Jasmine 85 is a fragrant *indica* variety which was developed at the International Rice Research Institute (IRRI) as IR841, from the cross IR262 × Khao Dawk Mali 105. It was released in the USA in 1989 as Jasmine 85 (Bollich, 1989; Asante, 2012). It was subsequently released as a commercial variety in Ghana in 2009 and for some time, was the most widely grown variety in Ghana because of its good taste, soft texture and fragrance (Asante et al., 2013; Ragassa et al., 2013). Jasmine 85 also has dehiscent anthers and non-exerted stigmas. The F_1_ progeny resulting from the cross between CRI-48 and Jasmine 85 exhibited anther indehiscence with exerted stigmas as observed in our previous study (Abebrese et al., 2018).

### 2.2 Developing the mapping population

**Figure 2:**
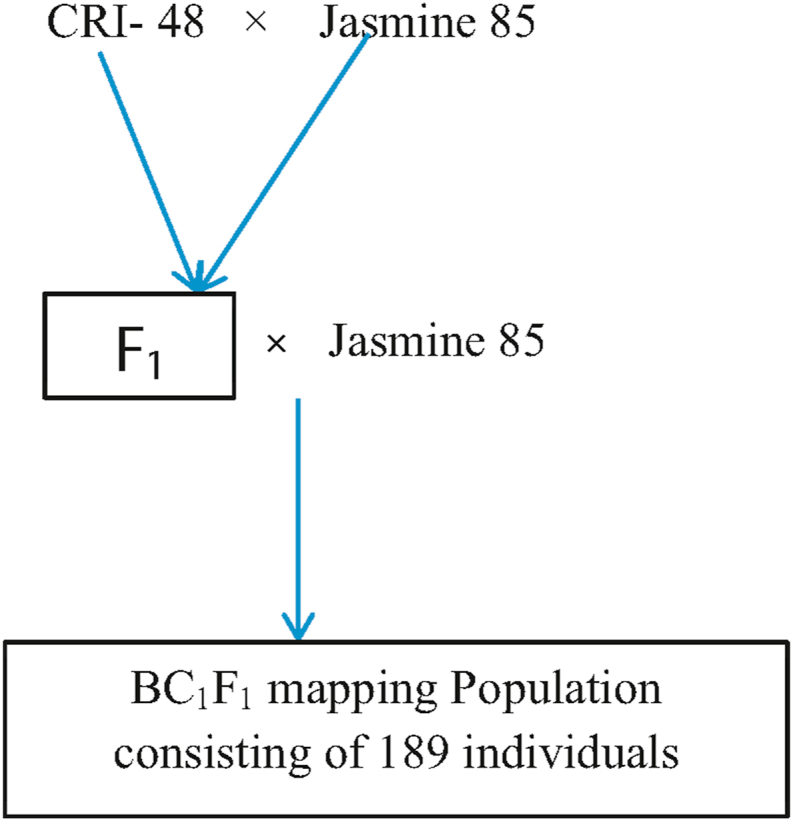
Crossing scheme used to generate the BC_1_F_1_ mapping population: Jasmine 85 was crossed to CR-48 as the male parent to generate the first filial generation (F_1_), the F_1_ was backcrossed to Jasmine 85 to generate 189 seeds which were used to raise 189 individuals used as the mapping population.

Jasmine 85 was crossed to CRI- 48 between July and October, 2013 at Nyankpala, Northern Ghana (09^0^ 24′ 17.8″ N, 000 ^0^ 57′ 57.0″ W, 143 m). The resultant F_1_ plants were raised in buckets. A single F_1_ plant was backcrossed to Jasmine 85 at the same location between July and October 2014. The 189 BC_1_F_1_ seeds were planted in buckets to raise 189 BC_1_F_1_ progenies which served as the mapping population for the present study (Figure 2).

### 2.3 Genotyping of the mapping population

Using a disc puncher, leaves (of 6mm diameter) were sampled from the two parents, four F_1_ plants and 189 BC_1_F_1_ plants three weeks after sowing and sent to LGC genomics, UK for DNA extraction and SNP genotyping. DNA extraction and KASP genotyping assay were carried out as described by Smith and Moughan (2015). The two parents were first screened with a total of 1885 SNP markers (LGC group) for polymorphism out of which 849 were polymorphic. Out of the 849 identified polymorphic markers, 246 evenly-spaced markers with known mapped positions were selected for genotyping the mapping population.

### 2.4 Phenotyping for anther indehiscence and stigma exertion

The phenotyping experiment was carried at Nyankpala, in the Guinea Savannah ecology of Northern Ghana (09^0^ 24′ 17.8″ N, 000 ^0^ 57′ 57.0″ W, 143 m). The seeds of the BC_1_F_1_ plants were pre-germinated in white tissue paper for four days and the resulting seedlings were nursed in buckets for 21 days followed by transplanting of one plant per 12 L bucket. Individual plants were provided with 8g of N.P.K. (15-15-15) fertilizer three weeks after transplanting, 4g of Ammonium sulphate at panicle initiation and watered whenever necessary. All other standard agronomic practices were followed as recommended. Individual plants were then phenotyped for the expression of anther indehiscence and stigma exertion. Dehiscence/indehiscence status of individual plants was scored by gently tapping panicles of individual plants at anthesis and visually observing extent of released pollen which was visible to the naked eye (Dartey, 2007). Absence of dehisced pollen was further checked with a hand lens to be sure that anthers remained indehiscent until drying up. Individual plants were scored for dehiscence/indehiscence of anthers and exerted/non-exerted of stigmas. Plants with dehiscent anthers and non-exerted stigmas were assigned zero (0) whereas their indehiscent counterparts with exerted stigmas were assigned one (1) for analysis.

### 2.5 Linkage map construction and QTL analysis

The genotyping data was used to construct a genetic linkage map for the CRI-48/Jasmine 85//Jasmine 85 BC_1_F_1_ population using QTL Network software v2.1 (Yang et al., 2007), a mixed model-based composite interval mapping (MCIM), based on default parameters of a 1000 permutation time, walk speed of 1cM, testing and filtration windows of 10cM each and a putative QTL detection at 0.05 significance level. MapChart Version 2.3 (Voorrips, 2002) was used for the construction of detailed linkage map showing the position of the QTL. The gene nomenclature followed that of McCouch et al. (1997) where a 2- or 3-letter abbreviation is followed by the number of chromosome on which the QTL is located and a terminal suffix, separated by a period, provides a unique identifier to distinguish multiple QTL on a single chromosome.

## 3 Results

### 3.1 Distribution of anther indehiscence and stigma exertion

**Figure 3:**
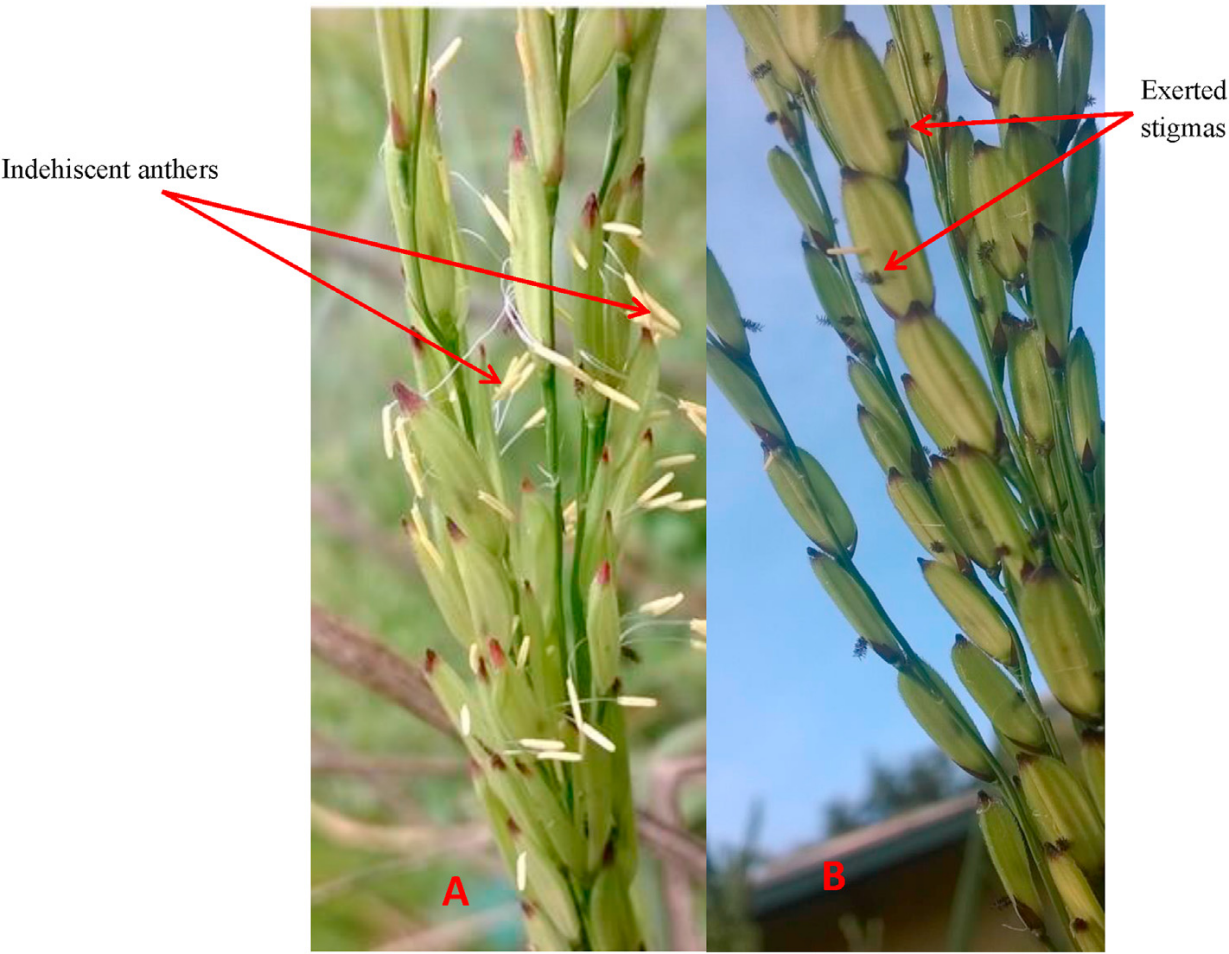
Anther indehiscence with exerted stigmas of the first filial generation (F_1_) between the CRI-48 and Jasmine 85 cross. Anthers fail to shed pollen till they wither (A). Stigmas exert outside the floret after indehiscent anthers wither (B).

The anther indehiscence trait was exhibited only by the F_1s_ and subsequent generations of the CRI-48/Jasmine 85 cross but not their individual parents. Both CRI-48 and Jasmine 85 had dehisced anthers with non-exerted stigmas. All the F_1_ plants from the CRI-48/Jasmine 85 cross exhibited anther indehiscence with exerted stigmas (Figure 3). The BC_1_F_1_ plants segregated for anther dehiscence/indehiscence and stigma exertion/non-exertion. Out of the 189 BC_1_F_1_ plants scored for the mapping study, 38 had dehiscent anthers whereas 151 had indehiscent anthers (Table 1). Thirty-eight (38) plants had their stigmas not exerted whereas 151 plants had their stigmas exerted (Table 1). Florets with indehiscent anthers always had their stigmas exerted outside the hull whilst stigmas were enclosed within the hull for florets with dehiscent anthers (Table 1). The two parents also differed in many agro-morphological traits including days to flowering, basal pigmentation and grain length. Whereas Jasmine 85 flowered within 85 days, CRI-48 flowered at 70 days. The BC_1_F_1_ plants showed variations and segregated for the various agro-morphological traits. Temperature at flowering did not have any effect on the expression of anther indehiscence.

**Table 1:** Anther indehiscence and stigma exertion status of BC_1_F_1_ plants.

Plant No.	Anther dehiscence status	Stigma exertion status	Plant No.	Anther dehiscence status	Stigma exertion status	Plant No.	Anther dehiscence status	Stigma exertion status	Plant No.	Anther dehiscence status	Stigma exertion status
Plant 1	Dehisce	Non exerted	Plant 26	Dehisce	Non exerted	Plant 51	Indehisce	Exerted	Plant 76	Indehisce	Exerted
Plant 2	Indehisce	Exerted	Plant 27	Dehisce	Non exerted	Plant 52	Indehisce	Exerted	Plant 77	Dehisce	Non exerted
Plant 3	Indehisce	Exerted	Plant 28	Indehisce	Exerted	Plant 53	Dehisce	Non exerted	Plant 78	Indehisce	Exerted
Plant 4	Dehisce	Non exerted	Plant 29	Indehisce	Exerted	Plant 54	Indehisce	Exerted	Plant 79	Indehisce	Exerted
Plant 5	Indehisce	Exerted	Plant 30	Dehisce	Non exerted	Plant 55	Indehisce	Exerted	Plant 80	Indehisce	Exerted
Plant 6	Dehisce	Non exerted	Plant 31	Dehisce	Non exerted	Plant 56	Indehisce	Exerted	Plant 81	Indehisce	Exerted
Plant 7	Indehisce	Exerted	Plant 32	Indehisce	Exerted	Plant 57	Indehisce	Exerted	Plant 82	Indehisce	Exerted
Plant 8	Indehisce	Exerted	Plant 33	Indehisce	Exerted	Plant 58	Dehisce	Non exerted	Plant 83	Indehisce	Exerted
Plant 9	Indehisce	Exerted	Plant 34	Indehisce	Exerted	Plant 59	Indehisce	Exerted	Plant 84	Indehisce	Exerted
Plant 10	Dehisce	Non exerted	Plant 35	Dehisce	Non exerted	Plant 60	Indehisce	Exerted	Plant 85	Indehisce	Exerted
Plant 11	Indehisce	Exerted	Plant 36	Dehisce	Non exerted	Plant 61	Indehisce	Exerted	Plant 86	Indehisce	Exerted
Plant 12	Indehisce	Exerted	Plant 37	Indehisce	Exerted	Plant 62	Dehisce	Non exerted	Plant 87	Indehisce	Exerted
Plant 13	Indehisce	Exerted	Plant 38	Indehisce	Exerted	Plant 63	Indehisce	Exerted	Plant 88	Indehisce	Exerted
Plant 14	Indehisce	Exerted	Plant 39	Dehisce	Non exerted	Plant 64	Indehisce	Exerted	Plant 89	Indehisce	Exerted
Plant 15	Indehisce	Exerted	Plant 40	Indehisce	Exerted	Plant 65	Indehisce	Exerted	Plant 90	Indehisce	Exerted
Plant 16	Indehisce	Exerted	Plant 41	Indehisce	Exerted	Plant 66	Dehisce	Non exerted	Plant 91	Indehisce	Exerted
Plant 17	Indehisce	Exerted	Plant 42	Indehisce	Exerted	Plant 67	Indehisce	Exerted	Plant 92	Indehisce	Exerted
Plant 18	Indehisce	Exerted	Plant 43	Indehisce	Exerted	Plant 68	Indehisce	Exerted	Plant 93	Indehisce	Exerted
Plant 19	Indehisce	Exerted	Plant 44	Dehisce	Non exerted	Plant 69	Indehisce	Exerted	Plant 94	Indehisce	Exerted
Plant 20	Indehisce	Exerted	Plant 45	Indehisce	Exerted	Plant 70	Indehisce	Exerted	Plant 95	Indehisce	Exerted
Plant 21	Dehisce	Non exerted	Plant 46	Indehisce	Exerted	Plant 71	Indehisce	Exerted	Plant 96	Dehisce	Non exerted
Plant 22	Indehisce	Exerted	Plant 47	Indehisce	Exerted	Plant 72	Indehisce	Exerted	Plant 97	Indehisce	Exerted
Plant 23	Dehisce	Non exerted	Plant 48	Indehisce	Exerted	Plant 73	Indehisce	Exerted	Plant 98	Indehisce	Exerted
Plant 24	Indehisce	Exerted	Plant 49	Dehisce	Non exerted	Plant 74	Indehisce	Exerted	Plant 99	Indehisce	Exerted
Plant 25	Indehisce	Exerted	Plant 50	Indehisce	Exerted	Plant 75	Indehisce	Exerted	Plant 100	Indehisce	Exerted
Plant No.	Anther dehiscence status	Stigma exertion status	Plant No.	Anther dehiscence status	Stigma exertion status	Plant No.	Anther dehiscence status	Stigma exertion status	Plant No.	Anther dehiscence status	Stigma exertion status
Plant 101	Indehisce	Exerted	Plant 126	Indehisce	Exerted	Plant 151	Indehisce	Exerted	Plant 176	Indehisce	Exerted
Plant 102	Indehisce	Exerted	Plant 127	Indehisce	Exerted	Plant 152	Indehisce	Exerted	Plant 177	Dehisce	Non exerted
Plant 103	Indehisce	Exerted	Plant 128	Indehisce	Exerted	Plant 153	Indehisce	Exerted	Plant 178	Indehisce	Exerted
Plant 104	Indehisce	Exerted	Plant 129	Indehisce	Exerted	Plant 154	Dehisce	Non exerted	Plant 179	Indehisce	Exerted
Plant 105	Indehisce	Exerted	Plant 130	Indehisce	Exerted	Plant 155	Indehisce	Exerted	Plant 180	Indehisce	Exerted
Plant 106	Dehisce	Non exerted	Plant 131	Indehisce	Exerted	Plant 156	Dehisce	Non exerted	Plant 181	Indehisce	Exerted
Plant 107	Dehisce	Non exerted	Plant 132	Indehisce	Exerted	Plant 157	Dehisce	Non exerted	Plant 182	Dehisce	Non exerted
Plant 108	Indehisce	Exerted	Plant 133	Indehisce	Exerted	Plant 158	Indehisce	Exerted	Plant 183	Indehisce	Exerted
Plant 109	Indehisce	Exerted	Plant 134	Indehisce	Exerted	Plant 159	Indehisce	Exerted	Plant 184	Indehisce	Exerted
Plant 110	Indehisce	Exerted	Plant 135	Dehisce	Non exerted	Plant 160	Indehisce	Exerted	Plant 185	Indehisce	Exerted
Plant 111	Indehisce	Exerted	Plant 136	Indehisce	Exerted	Plant 161	Indehisce	Exerted	Plant 186	Indehisce	Exerted
Plant 112	Dehisce	Non exerted	Plant 137	Indehisce	Exerted	Plant 162	Dehisce	Non exerted	Plant 187	Indehisce	Exerted
Plant 113	Indehisce	Exerted	Plant 138	Dehisce	Non exerted	Plant 163	Indehisce	Exerted	Plant 188	Indehisce	Exerted
Plant 114	Indehisce	Exerted	Plant 139	Indehisce	Exerted	Plant 164	Indehisce	Exerted	Plant 189	Dehisce	Non exerted
Plant 115	Indehisce	Exerted	Plant 140	Indehisce	Exerted	Plant 165	Indehisce	Exerted			
Plant 116	Indehisce	Exerted	Plant 141	Indehisce	Exerted	Plant 166	Indehisce	Exerted			
Plant 117	Indehisce	Exerted	Plant 142	Indehisce	Exerted	Plant 167	Dehisce	Non exerted			
Plant 118	Dehisce	Non exerted	Plant 143	Dehisce	Non exerted	Plant 168	Indehisce	Exerted			
Plant 119	Indehisce	Exerted	Plant 144	Dehisce	Non exerted	Plant 169	Indehisce	Exerted			
Plant 120	Indehisce	Exerted	Plant 145	Indehisce	Exerted	Plant 170	Indehisce	Exerted			
Plant 121	Indehisce	Exerted	Plant 146	Indehisce	Exerted	Plant 171	Indehisce	Exerted			
Plant 122	Indehisce	Exerted	Plant 147	Indehisce	Exerted	Plant 172	Indehisce	Exerted			
Plant 123	Indehisce	Exerted	Plant 148	Indehisce	Exerted	Plant 173	Indehisce	Exerted			
Plant 124	Indehisce	Exerted	Plant 149	Indehisce	Exerted	Plant 174	Indehisce	Exerted			
Plant 125	Indehisce	Exerted	Plant 150	Indehisce	Exerted	Plant 175	Dehisce	Non exerted			

### 3.2 Genetic analysis and QTL detection

**Figure 4:**
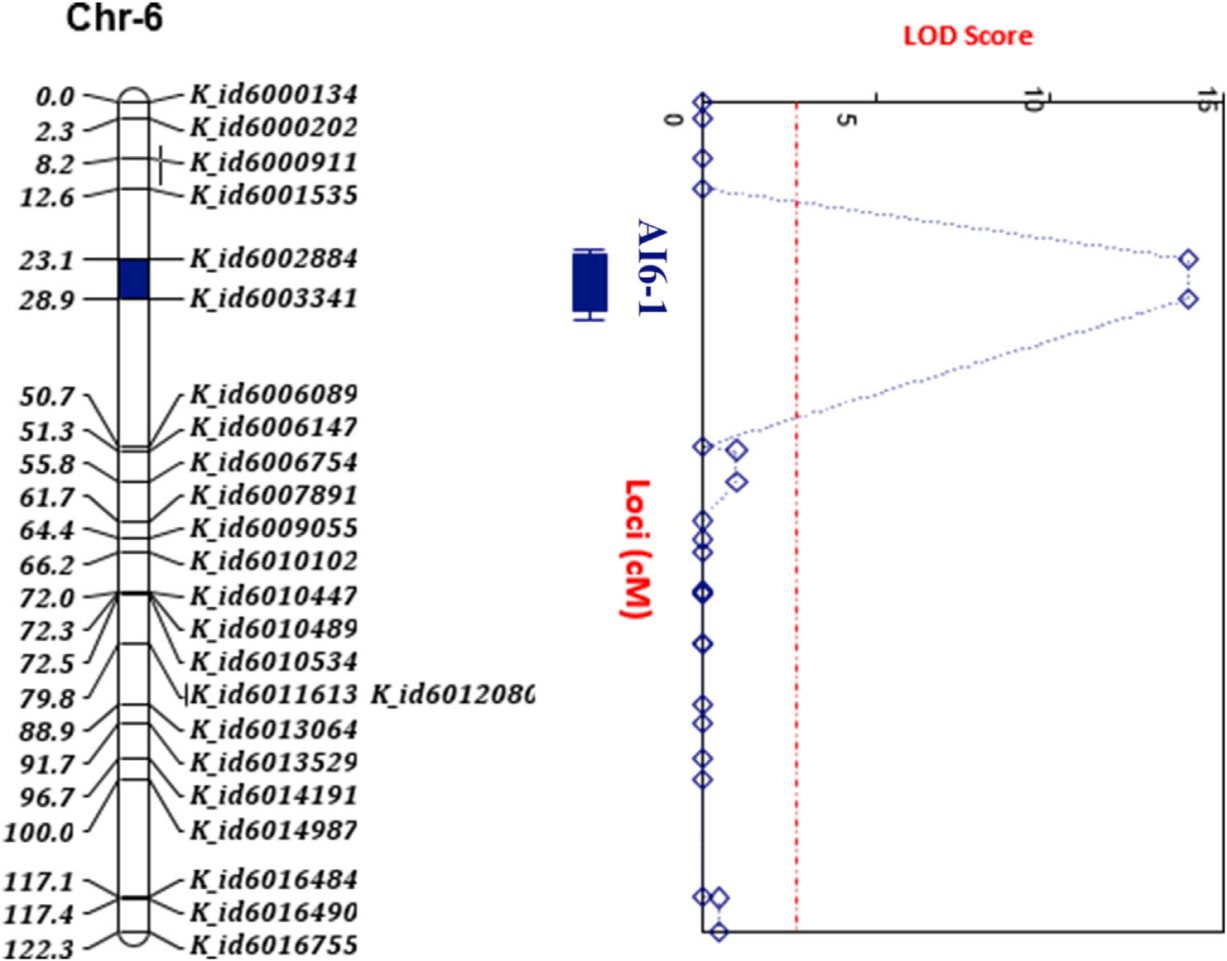
Genetic linkage map showing the locus (*AI6-1*) mapped for anther indehiscence with exerted stigmas on chromosome 6 between SNP markers K_ID6002884 and K_ID6003341.

A genetic linkage map with 12 linkage groups corresponding to the 12 gametic rice chromosomes was constructed, spanning a total length of 1520.2 cM at an average marker interval of 6.18 cM (Table 2) using 246 markers. Chromosome 1 was the longest (179.4 cM) and had 40 markers with an average marker density of 4.49 cM. Chromosome 9 spanned 98.6 cM and was the shortest with average marker density of 7.58 cM. Summary of marker positions on the genetic linkage map is presented in Table 2. A single locus *(AI6-1)* was mapped at 27.4 cM on chromosome 6 for anther indehiscence with exerted stigmas. This locus was flanked by K_ID6002884 and K_ID6003341 within a range of 23.1–28.9 cM (Table 3; Figure 4). The allele at this locus was contributed by the CRI-48 parent which has *Oryza glaberrima* ancestry (Table 3).

**Table 2:** Summary of genetic linkage map for the 246 SNP markers.

Chromosome	Length (cM)	Number of SNP makers	Average marker density (cM)
1	179.4	40	4.49
2	142	25	5.68
3	160.4	22	7.29
4	114.6	22	5.21
5	132.1	23	5.74
6	122.3	24	5.1
7	108	22	4.91
8	130.2	17	7.66
9	98.6	13	7.58
10	100.9	11	9.17
11	114.4	14	8.17
12	117.3	14	8.38
Total/Average	1520.2	246	6.18

**Table 3:** Information on the locus identified for anther indehiscence with exerted stigmas.

Locus	Chr.	Interval	position	range	A	SE	P-Value	Source of allele
*AI6-1*	6	K_ID6002884-K_ID6003341	27.4	23.1–28.9	-0.8388	0.0793	0.00001	CRI-48

## 4 Discussion

This study was set out to preliminarily map the chromosomal locations controlling anther indehiscence with exerted stigmas in rice for further studies on fine mapping and cloning the underlining gene (s). The underlying gene(s) could possibly be manipulated through marker assisted selection (MAS) or genetic engineering to develop male sterile rice lines with enhanced outcrossing for future hybrid rice seed production. The study followed the bi-parental mapping procedure.

Diverse parents are *in vogue* recommended for bi-parental QTL mapping studies to enable high marker polymorphism detection and adequate variation within the trait of interest (Collard et al., 2008; Jones et al., 1997, 2009). The presence of 849 polymorphic markers, representing 45% of the total 1885 SNP markers from the initial polymorphism survey suggests that the two parents were different in most of their genomic regions. This was likely because Jasmine 85 (the male parent) is an *indica* variety whereas the CRI-48 parent (the female parent) is from an interspecific *japonica*/NERICA cross with *O. glaberrima* parentage (Somado et al., 2008). A high-density genetic linkage map with evenly distributed markers is a prerequisite for identifying chromosomal regions that contain genes of interest using QTL analysis (McCouch and Doerge, 1995; Bernardo, 2008; Collard et al., 2008). A map length of 1520.2 cM generated from the 246 evenly distributed SNP markers was similar in length to linkage maps constructed using simple sequence repeat (SSR), restriction fragment length polymorphism (RFLPs) and amplified fragment length polymorphism (AFLPs) markers (Lanceras et al., 2000; Temnykh et al., 2000; Collard et al., 2008). An average marker density of 6.18 cM for the constructed map was appropriate for initial QTL detection. Bernardo (2008) recommended average marker density of <10 cM for such purposes.

Expression of anther indehiscence only by the F_1_s but not their individual parents suggests that the trait might be as a result of complementary genes from the two parents. Different modes of inheritance have been reported for anther indehiscence from different cross combinations (Sano, 1986; Maekaewa et al., 1997; Dartey, 2007). Our earlier study (Abebrese et al., 2018) found anther indehiscence with exerted stigmas in the CRI-48/Jasmine 85 cross to conform to the three complementary genes mode of inheritance reported by Dartey (2007). However, using genome-wide SNP markers, a single locus (*AI6-1*) was mapped for anther indehiscence with exerted stigmas in this current study. It could be that, the three complementary genes suggested by conventional genetic analysis are in a cluster. Fine mapping using denser molecular markers could reveal more in this direction. Segregation of anther indehiscence in the mapping population was skewed and did not fit into any of the earlier reported ratios (Sano, 1986; Maekawa et al., 1997; Dartey, 2007). Failure of the segregating pattern of the mapping population to conform to the 7:1 (indehiscence: dehiscence) mode of inheritance reported earlier could be due to the smaller population size. Also, hybridity of individual BC_1_F_1_ plants was mostly established by phenotypically examining the plants to confirm combination of unique traits of the two parents. Few plants which lacked such clear trait combinations were discarded. Such minor selection might have also contributed to the segregation distortion observed in the mapping population.

The locus for anther indehiscence with exerted stigmas in this study was mapped to 27.4 cM on chromosome 6. This locus was flanked by K_ID6002884 and K_ID6003341 within a marker interval of 23.1–28.9 cM Zhu et al. (2004) identified a rice (*Oryza sativa* L. cv Nipponbare) recessive mutant, anther indehiscence (*aid1*) gene, through the reverse genetics approach (a two-element iAc/Ds transposon-tagging system), showing partial to complete spikelet sterility. The *aid1* gene which was mapped to 13.5 cM (124,000–140,000 bp) on chromosome 6 is about 13.9 cM away from the locus mapped in this present study. Among the several QTLs reported for stigma exertion of rice (Uga et al., 2003; Miyata et al., 2007; Yan et al., 2009; Lou et al., 2014), two (*qPDES-6* and *qPES-6*) have been mapped on chromosome 6 (Lou et al., 2014). These two QTLs were flanked by simple sequence repeat (SSR) markers RM8225 and RM225 within an interval of 26.2–54.1 cM (3,416,523–9,309,118 bp, Nipponbare sequence 2009, www.gramene.org) on chromosome 6. The locus for anther indehiscence with exerted stigmas in this present study which was mapped within 23.1–28.9 cM is in the range reported by Lou et al. (2014). Florets with indehiscent anthers always had their stigmas exerted outside the hull whereas stigmas were enclosed within the hull for florets with dehisced anthers. Anther indehiscence always co-segregated with stigma exertion in a 964 BC_1_F_1_ segregating population reported by Abebrese et al. (2018) and that of a 517 reported by Dartey (2007). Therefore, it seems the single locus (*AI6-1*) controls anther indehiscence and stigma exertion pleiotropically. The *aid1* gene reported by Zhu et al. (2004) had a pleiotropic effect on tillering and flowering time. Presence of pleiotropy could aid in manipulating the two traits together to design a useful male sterility system with enhanced outcrossing.

Review of literature suggests two sources of anther indehiscence genes. Anther indehiscence could originate from a single rice genotype or species (Cheng and Huang 1980; Sano, 1986; Li et al., 2011). For instance, Sano (1986) suggested a dominant gene (*W020*) from *O. glaberrima* as responsible for anther indehiscence. Cheng and Huang (1980) also traced anther indehiscence genes to *O. rufipogon.* Alternatively, anther indehiscence could also be as a result of complementary action of genes from two genotypes or species (Maekaewa et al., 1997; Dartey, 2007). Maekaewa et al. (1997) suggested that anther indehiscence is controlled by complementary action of three dominant genes. In their study, cv. Silewah (one of the parents for their mapping population) putatively had one of the three genes and cv. Hayakogane (the other parent) had the other two. Dartey (2007) also postulated involvement of three complementary genes to control anther indehiscence. Anthers dehisce if all three genes exist in the homozygous state, but indehiscence would result if one, two or all three genes exist in the heterozygous state. The allele at the mapped locus for this current study was contributed by the CR-48 parent. The CRI-48 has a *glaberrima* ancestry from its NERICA parent. The source of the anther indehiscence gene(s) could possibly be traced to this *glaberrima* parent. Anther indehiscence has also been reported as a common phenomenon in *glaberrima*-*sativa* crosses and was attributed to chromosomal aberrations (Sano, 1986).

## 5 Conclusion

The study identified a single mapped locus between SNP markers K_ID6002884 and K_ID6003341 on chromosome 6 for anther indehiscence with exerted stigmas. The allele at this locus was contributed by the CRI-48 parent which has *Oryza glaberrima* ancestry. We suggest that this locus controls anther indehiscence and stigma exertion through pleiotropic gene action or the three complementary genes might be in a cluster. Fine mapping with denser molecular markers could help uncover the underlying gene(s).

## Declarations

### Author contribution statement

Samuel Oppong Abebrese: Conceived and designed the experiments; Performed the experiments; Analyzed and interpreted the data; Contributed reagents, materials, analysis tools or data; Wrote the paper.

Nana Kofi Abaka Amoah: Performed the experiments; Analyzed and interpreted the data; Contributed reagents, materials, analysis tools or data; Wrote the paper.

Paul Kofi Ayirebi Dartey: Conceived and designed the experiments; Performed the experiments; Analyzed and interpreted the data; Contributed reagents, materials, analysis tools or data.

Isaac Kofi Bimpong: Analyzed and interpreted the data; Contributed reagents, materials, analysis tools or data.

Richard Akromah: Conceived and designed the experiments; Analyzed and interpreted the data; Contributed reagents, materials, analysis tools or data.

Vernon Edward Gracen; Samuel Kwame Offei; Eric Yirenkyi Danquah: Conceived and designed the experiments; Contributed reagents, materials, analysis tools or data.

### Funding statement

This work was supported by 10.13039/100013718Alliance for a Green Revolution in Africa (AGRA) through the West Africa Centre for Crop Improvement (WACCI), 10.13039/501100005601University of Ghana

### Data availability statement

Data included in article/supplementary material/referenced in article.

### Competing interest statement

The authors declare no conflict of interest.

### Additional information

No additional information is available for this paper.

## References

[bib1] Abebrese S.O., Dartey P.K.A., Akromah R., Gracen V.E., Offei S.K., Danquah E.Y. (2018). Genetics of anther indehiscence with exerted stigmas and its application in hybrid rice breeding. J. Crop Improv..

[bib2] Asante M.D. (2012). Genetic Analysis of Grain Quality Traits in rice.

[bib3] Asante M.D., Owusu B.A., Acheampong G.K., Offei S.K., Gracen V., Adu-Dapaah H., Danquah E.Y. (2013). Farmer and consumer preferences for rice in the Ashanti region of Ghana: implications for rice breeding in West Africa. J. Plant Breed Crop Sci..

[bib4] Bakti C., Tanaka J. (2019). Detection of dominant QTLs for stigma exsertion ratio in rice derived from *Oryza rufipogon* accession ‘W0120’. Breed Sci..

[bib5] Bernardo R. (2008). Molecular markers and selection for complex traits in plants: learning from the last 20 years. Crop Sci..

[bib6] Bollich C.N. (1989). Release of a new rice cultivar Jasmine 85 in the USA. Int. Rice Res. Newsl..

[bib7] Breseghello F., Coelho A.S.G. (2013). Traditional and modern plant breeding methods with examples in rice (*Oryza sativa* L.). J. Agric. Food Chem..

[bib8] Cardarelli M., Costantino P. (2018). An auxin switch for male fertility. Nat. Plants.

[bib9] Chang Z., Chen Z., Wang N., Xie G., Lu J., Yan W. (2016). Construction of a male sterility system for hybrid rice breeding and seed production using a nuclear male sterility gene. Proc. Natl. Acad. Sci. Unit. States Am..

[bib10] Cheng Y., Huang C. (1980). Studies into Cytoplasmic- genetic male sterility of cultivated rice (*Oryza sativa* L): morphological-historical investigation on functional male sterility. Chin. J. Agric. Res..

[bib11] Collard B.C., Jahufer M.Z.Z., Brouwer J.B., Pang E.C.K. (2005). An introduction to markers, quantitative trait loci (QTL) mapping and marker-assisted selection for crop improvement: the basic concepts. Euphytica.

[bib12] Collard B.C., Cruz C.M.V., McNally K.L., Virk P.S., Mackill D.J. (2008). Rice molecular breeding laboratories in the genomics era: current status and future considerations. Int. J. Plant Genom..

[bib13] Dartey P.K.A. (2007). Genic control of Anther indehiscence in rice. Indian J. Crop Sci..

[bib14] Estornell L.H., Landberg K., Cierlik I., Sundberg E. (2018). SHI/STY genes affect pre-and post-meiotic anther processes in auxin sensing domains in Arabidopsis. Front. Plant Sci..

[bib15] FAO (2017). Rice Market Monitor (RMM).

[bib16] Fischer R.A., Byerlee D., Edmeades G.O. (2014). Crop Yields and Global Food Security: Will Yield Increase Continue to Feed the World? ACIAR Monograph No. 158.

[bib17] Goldberg R.B., Beals T.P., Sanders P.M. (1993). Anther development: basic principles and practical applications. Plant Cell.

[bib18] Guimaraes E.P., Carena M.J. (2009). Rice breeding. Cereals, the Banks and the Italian Economy.

[bib19] IRRI (1997). Hybrid rice Breeding Manual.

[bib20] IRRI (2010). Global Rice Science Partnership (GRISP) Full Proposal.

[bib21] Jones N., Ougham H., Thomas H. (1997). Markers and mapping: we are all geneticists now. New Phytol..

[bib22] Jones N., Ougham H., Thomas H., Pasakinskiene I. (2009). Markers and mapping revisited: finding your gene. New Phytol..

[bib23] Keijzer C. (1987). The processes of anther dehiscence and pollen dispersal. I. The opening mechanism of longitudinally dehiscing anthers. New Phytol..

[bib24] Khan M.H., Dar Z.A., Dar S.A. (2015). Breeding strategies for improving rice yield—a review. Agric. Sci..

[bib25] Khush G.S. (2005). What it will take to feed 5.0 rice consumers in 2030. Plant Mol. Biol..

[bib26] Kobayashi K., Matsui T., Murata Y., Yamamoto M. (2011). Percentage of dehisced thecae and length of dehiscence control pollination stability of rice cultivarsat high temperatures. Plant Prod. Sci..

[bib27] Lanceras J.C., Huang Z.-L., Naivikul O., Vanavichit A., Ruanjaichon V., Tragoonrung S. (2000). Mapping of genes for cooking and eating qualities in Thai jasmine rice (KDML105). DNA Res..

[bib28] Li F., Liu F.H., Morinaga D., Zhao Z. (2011). A new gene for hybrid sterility from a cross between Oryza sativa and O. glaberrima. Plant Breed..

[bib29] Li P., Feng F., Zhang Q., Chao Y., Gao G., He Y. (2014). Genetic mapping and validation of quantitative trait loci for stigma exertion rate in rice. Mol. Breed..

[bib30] Ling S., Chen C., Wang Y., Sun X., Lu Z., Ouyang Y., Yao J. (2015). The mature anther-preferentially expressed genes are associated with pollen fertility, pollen germination and anther dehiscence in rice. BMC Genom..

[bib31] Lou J., Yue G.H., Yang W.Q., Mei H.W., Luo L.J., Lu H.J. (2014). Mapping QTLs influencing stigma exertion in rice. Bulgarian J. Agric. Sci..

[bib32] Ma H. (2005). Molecular genetic analyses of microsporogenesis and microgametogenesis in flowering plants. Annu. Rev. Plant Biol..

[bib33] Maekawa M., Inukai T., Shinbashi N. (1997). Genic analysis of hybrid sterility caused by anther indehiscence between distantly related rice varieties. Euphytica.

[bib34] Matsui T., Omasa K., Horie T. (1999). Mechanism of anther dehiscence in rice (Oryza sativa L.). Ann. Bot..

[bib35] McCouch S.R., Doerge R.W. (1995). QTL mapping in rice. Trends Genet..

[bib36] McCouch S.R., Cho Y., Yano M., Paul E., Blinstruub M. (1997). Report on QTL nomenclature. Rice Genetics Newsletter.

[bib37] McCouch S.R., Zhao K., Wright M., Tung C.-W., Ebana K., Thomson M., Reynolds A., Wang D., DeClerck G., Ali M.L., McClung A., Eizenga G., Bustamante C. (2010). Development of genome-wide SNP assays for rice. Breed Sci..

[bib38] McCouch S., Wing R.A., Semon M., Vanuprasad R., Atlin G., Sorrells M.E., Jannink J., Wopereis M.C.S., Johnson D.E., Ahmadi N., Tollens E., Jalloh A. (2013). Making rice genomics work for Africa. Realizing Africa's Rice Promise.

[bib39] Miyata M., Yamamoto T., Komori T., Nitta N. (2007). Marker assisted selection and evaluation of the QTL for stigma exertion under japonica rice genetic background. Theor. Appl. Genet..

[bib40] Moon S., Jung K.H. (2020). First steps in the successful fertilization of rice and arabidopsis: pollen longevity, adhesion and hydration. Plants.

[bib41] Muthayya S., Sugimoto J.D., Montgomery S., Maberly G.F. (2014). An overview of global rice production, supply, trade, and consumption. Ann. N. Y. Acad. Sci..

[bib42] Nadeem M.A., Nawaz M.A., Shahid M.Q., Doğan Y., Comertpay G., Yıldız M. (2018). DNA molecular markers in plant breeding: current status and recent advancements in genomic selection and genome editing. Biotechnol. Biotechnol. Equip..

[bib43] Oka H.I., Khush G.S., Toenniessen G.H. (1991). Genetic diversity of wild and cultivated rice. Rice Biotechnology.

[bib44] Peng Y.J., Shih C.F., Yang J.Y., Tan C.M., Hsu W.H., Huang Y.P. (2013). A RING-type E 3 ligase controls anther dehiscence by activating the jasmonate biosynthetic pathway gene defective in anther dehiscence 1 in A rabidopsis. Plant J..

[bib45] Ragassa C., Dankyi A., Acheampong P., Wiredu A.N., Chapo-to A., Asamoah M., Tripp R. (2013). Patterns of adoption of improved rice technologies in Ghana.

[bib46] Sano Y. (1986). Sterility barriers between *Oryza sativa* and *O. Glaberrima*. Rice Genetics, Proceedings of the International Rice Genetics Symposium 27–31 May 1985.

[bib47] Seck P.A., Diagne A., Mohanty S., Wopereis M.C.S. (2012). Crops that feed the world 7: rice. Food Secur..

[bib48] Seck P.A., Toure A.A., Coulibali J.Y., Diangne A., Wopereis M.C.S., Wopereis M.C.S., Johnson D.E., Ahmadi N., Tollens E., Jalloh A. (2013). Africa's rice economy before and after the 2008 rice crises. Realizing Africa's Rice Promise.

[bib49] Semagn K., Bjornstad A., Ndjiondjop M.N. (2006). An overview of molecular marker methods for plants. Afr. J. Biotechnol..

[bib50] Semagn K., Babu R., Hearne S., Olsen M. (2014). Single nucleotide polymorphism genotyping using Kompetitive Allele Specific PCR (KASP): overview of the technology and its application in crop improvement. Mol. Breed..

[bib51] Smith S.M., Moughan P.J., Batley J. (2015). SNP genotyping using KASPar assays. Plant Genotyping: Methods and Protocols.

[bib52] Somado E.A., Guei R.G., Keya S.O. (2008). NERICA: the New rice for Africa–A Compendium.

[bib53] Steele K.A., Quinton-Tulloch M.J., Amgai R.B., Dhakal R., Khatiwada S.P., Vyas D. (2018). Accelerating public sector rice breeding with high-density KASP markers derived from whole genome sequencing of indica rice. Mol. Breed..

[bib54] Takano-Kai N., Doi K., Yoshimura A. (2011). GS3 participates in stigma exsertion as well as seed length in rice. Breed Sci..

[bib55] Tamaru N. (1991). Frequency of pollen grain stainability with I-KI solution and features of genetic male sterile lines in rice after anthesis. J. Hokkaido Univ. Educ..

[bib56] Temnykh S., Park W.D., Ayres N., Cartinhour S., Hauck N., Lipovich L., Cho Y.G., Ishii T., McCouch S.R. (2000). Mapping and genome organization of microsatellite sequences in rice (Oryza sativa L.). Theor. Appl. Genet..

[bib57] Thangasamy S., Guo C., Chuang M., Lai M., Chen J., Jauh G. (2011). Rice SIZ1, a SUMO E3 ligase, controls spikelet fertility through regulation of anther dehiscence. New Phytol..

[bib58] Toure A., Haussmann B.I.G., Jones N., Thomas H., Ougham H., Haussmann B.I., Geiger G., H. H, Hess D.E., Hash C.T., Bramel-Cox P. (2000). Construction of a genetic map, mapping of major genes, and QTL analysis. Application of Molecular Markers in Plant Breeding. Training Manual for a Seminar Held at IITA, Ibadan, Nigeria, from 16-17 August 1999. International Crops Research Institute for the Semi-arid Tropics (ICRISAT), Patancheru 502 324.

[bib59] Uga Y., Fukuta Y., Cai H.W., Iwata H., Ohsawa R., Morishima H., Fujimura T. (2003). Mapping QTLs influencing rice floral morphology using recombinant inbred lines derived from a cross between *Oryza sativa* L. and *Oryza rufipogon* Griff. Theor. Appl. Genet..

[bib60] Virmani S.S. (1994). Heterosis and Hybrid Rice Breeding.

[bib61] Virmani S.S., Sun Z.X., Mou T.M., Jauhar Ali A., Mao C.X. (2003). Two-line Hybrid rice Breeding Manual.

[bib62] Voorrips R.E. (2002). MapChart Version 2.3 Software of graphical presentation of linkage maps and QTLs. J. Hered..

[bib63] Wilson Z.A., Song J., Taylor B., Yang C. (2011). The final split: the regulation of anther dehiscence. J. Exp. Bot..

[bib64] Xu S., Zheng Y., Liu Y., Guo X., Tan Y., Qian Q. (2019). Identification of a major quantitative trait locus and its candidate underlying genetic variation for rice stigma exsertion rate. Crop J..

[bib65] Yan G.W., Li Y., Hesham A., Agrama H.A., Luo D., Gao F., Lu X., Ren G. (2009). Association mapping of stigma and spikelet characteristics in rice (*Oryza sativa L.*). Mol. Breed..

[bib66] Yang J., Zhu J., Williams R.W. (2007). Mapping the genetic architecture of complex traits in experimental populations. Bioinformatics.

[bib67] Yang G., Chen S., Chen L., Sun K., Huang C., Zhou D. (2019). Development of a core SNP arrays based on the KASP method for molecular breeding of rice. Rice.

[bib68] Young N.D. (1994). Constructing a plant genetic linkage map with DNA markers. DNA-based Markers in Plants.

[bib69] Zhou S., Wang Y., Li W., Zhao Z., Ren Y., Wang Y. (2011). Pollen semi-sterility1 encodes a kinesin-1–like protein important for male meiosis, anther dehiscence, and fertility in rice. Plant Cell.

[bib70] Zhu Q.H., Ramm K., Shivakkumar R., Dennis E.S., Upadhyaya N.M. (2004). The anther indehiscence gene encoding a single MYB Domain protein is involved in anther development in rice. Plant Physiol..

